# A Case of Non-Irradiated Balloon Cell Melanoma of the Choroid: Expanding the Morphological Spectrum of Primary Uveal Melanomas

**DOI:** 10.3390/diagnostics12030642

**Published:** 2022-03-05

**Authors:** Maria Failla, Rosario Caltabiano, Antonio Longo, Andrea Russo, Michele Reibaldi, Teresio Avitabile, Eliana Piombino, Cristina Colarossi, Lorenzo Colarossi, Elena Tirrò, Paolo Vigneri, Pietro Valerio Foti, Giuseppe Broggi

**Affiliations:** 1 Department of Medical, Surgical Sciences and Advanced Technologies “G.F. Ingrassia”, Anatomic Pathology, University of Catania, 95123 Catania, Italy; rosario.caltabiano@unict.it (R.C.); giuseppe.broggi@gmail.com (G.B.); 2 Pathology Unit, Department of Experimental Oncology, Mediterranean Institute of Oncology, 95029 Viagrande, Italy; eliana.piombino@grupposamed.com (E.P.); cristina.colarossi@grupposamed.com (C.C.); lorenzo.colarossi@grupposamed.com (L.C.); 3 Eye Clinic, Department of Ophthalmology, University of Catania, 95123 Catania, Italy; antlongo@unict.it (A.L.); andrearusso2000@hotmail.com (A.R.); mreibaldi@libero.it (M.R.); t.avitabile@unict.it (T.A.); 4 Department of Surgical Sciences, Eye Clinic Section, University of Turin, 10126 Turin, Italy; 5 Center of Experimental Oncology and Hematology, A.O.U. Policlinico “G. Rodolico—San Marco”, 95123 Catania, Italy; ele_tir@yahoo.it (E.T.); vigneri.p@unict.it (P.V.); 6 Department of Surgical, Oncological and Stomatological Sciences, University of Palermo, 90127 Palermo, Italy; 7 Radiology I Unit, Department of Medical Surgical Sciences and Advanced Technologies G.F. Ingrassia, University Hospital Policlinico “G. Rodolico-San Marco”, 95123 Catania, Italy; pietrofoti@hotmail.com

**Keywords:** uveal melanoma, balloon cell, eye tumor, non-irradiated melanoma of the choroid

## Abstract

Uveal melanoma (UM) is the most common primary intraocular tumor in adults and usually has a very poor prognosis. Histologically, UMs have been classified in epithelioid cell type, spindle cell type, and mixed cell type. Balloon cells are large pale cells that contain small, hyperchromatic, central nuclei with vesiculated, clear, and lipid-rich cytoplasm. A balloon cell morphology is infrequently observed in naevi and even less frequently in malignant melanomas of the skin, conjunctiva, ciliary body and choroid. In this regard, UMs that exhibit balloon cell features are generally those previously treated with proton beam irradiation and then enucleated, rather than those that directly underwent primary surgery. To the best of our knowledge, very few cases of primary UM showing extensive balloon cell morphology have been reported in scientific literature to date. We herein present an unusual case of primary UM with diffuse balloon cell changes in a 69-year-old woman.

## 1. Introduction

Uveal melanoma (UM) arises from melanocytes within the choroid, the ciliary body, and the iris of the eye, and, despite being generally considered a rare neoplasm, is the most common primary intraocular tumor in adults [1]. One of the differences between cutaneous and UM is the strong tendency of the latter to selectively metastasize to the liver; in fact, up to 50% of patients with primary UM will ultimately develop distant metastasis with a liver involvement in up to 90% of individuals [1]. The fact that cutaneous and UM are two biologically distinct tumors is also confirmed by their different molecular landscape: whereas cutaneous melanoma commonly harbors mutations mainly affecting the mitogen-activated protein kinase (MAPK) pathway and codon-600 of *BRAF* kinase, *GNAQ*, *GNA11*, *SF3B1*, *EIF1AX*, and *BAP1* are the most frequently mutated genes in UM [2].

The anatomical location most frequently affected by UM is the choroid, then the ciliary bodies, and lastly, the iris. Iris melanomas (IMs) generally have a better prognosis than the others, possibly because IMs are usually visible from the outside of the eye and thus receive an early diagnosis [3]. Most UMs, if not all, originate from a transformed nevus. [4]. Therapeutic options include proton beam radiotherapy, gamma knife stereotactic radiosurgery, transpupillary thermotherapy, laser photocoagulation, local surgical resection, and enucleation [5]. Small (1.5–2.4 mm height and 5–16 mm diameter) and medium-sized (2.5–10 mm apical height and ≤16 mm diameter) tumors are mainly treated by radiotherapy, whereas large tumors, especially if locally advanced, are mostly treated by enucleation [6]. The latest findings in ocular melanoma biology have led to research on immunotherapies, anti-angiogenic agents, and targeted therapies, including kinase inhibitors such as sorafenib, sunitinib, and imatinib [6]. Histologically, UMs have been classified in epithelioid cell type, spindle cell type, and mixed cell type. Balloon cells are large pale cells that contain small, hyperchromatic, central nuclei with vesiculated, clear, and lipid-rich cytoplasm. A balloon cell morphology is rarely observed in naevi and even less frequently in malignant melanomas of the skin, conjunctiva, ciliary body, and choroid; on the other hand, balloon cell formation is a common finding in UMs previously treated with proton beam irradiation and then enucleated, alongside other degenerative changes including pyknotic nuclei, tumor cell necrosis, vacuolization, vascular obstruction, and fibrosis of the tumor stroma with an accumulation of pigmented macrophages [7,8]. Therefore, it can be said that UMs that exhibit balloon cell features are generally those that have been previously treated with proton beam irradiation and then enucleated, rather than those that directly underwent primary surgery [9], as confirmed by different authors [7,8]. To the best of our knowledge, very few cases of primary UM showing extensive balloon cell morphology have been reported in scientific literature to date [10,11]. We herein present an unusual case of primary UM with diffuse balloon cell changes in a 69-year-old woman.

## 2. Materials and Methods

### 2.1. DNA Extraction and Real Time PCR Analysis

Genomic DNA was isolated from 8 FFPE sections 5 microns thick. After an initial step with deparaffinization solution (Qiagen, Hilden, Germany) used to eliminate paraffin from the sample, the DNA was extracted with a High Pure FFPET DNA isolation kit (Roche LifeScience, Basel, Switzerland) following the manufacturer’s instructions. DNA concentration was detected with Quantus Fluorometer utilizing QuantiFluor dsDNA kit (Promega, Madison, WI, USA). A total of 300 ng of DNA were used for quantitative real-time PCR. A mutational analysis of NRAS and BRAF genes was carried out with EasyPgX kits (Diatech Pharmacogenetics, Ancona, Italy) according to the manufacturer’s recommendations. The evaluation of the mutational status of NRAS on exon 2 (codons 12,13), exon 3 (codons 59, 61), and exon 4 (codons 61, 117) was performed using 8 specific oligos. The mutational status on codon 600 (V600E, V600K, V600D, V600R) of BRAF was analyzed using 4 specific oligo mixes. A total of 25 ng of genomic DNA was used as a template for each reaction. Real-time PCR was performed using AriaDx instrument (Agilent Technologies, Santa Clara, CA, USA).

### 2.2. DNA Extraction and Next Generation Sequencing (NGS)

DNA was extracted from the FFPE sample using a Gene Read DNA FFPE kit (Qiagen), and its yield was assessed by a dsDNA HS Assay kit on a Qubit 3.0 Fluorometer (both from ThermoFisher Scientific, Waltham, MA, USA). A total of 10 ng input DNA was used for next-generation sequencing (NGS) analysis. The library was generated with an Ion Ampliseq library kit plus and the Ion Ampliseq cancer HotSpot Panel v2 primer pool (both from Thermo Fisher Scientific, Waltham, MA, USA). An Ion Chef System was then used for automated template preparation and loading the sequencing chip, and sequencing was carried out on an Ion GeneStudio S5 Plus System (both from ThermoFisher Scientific, Waltham, MA, USA). Ion Reporter v5.18.2.0 (ThermoFisher Scientific, Waltham, MA, USA) was employed for single nucleotide variant annotations.

## 3. Case Presentation

**Figure 1:**
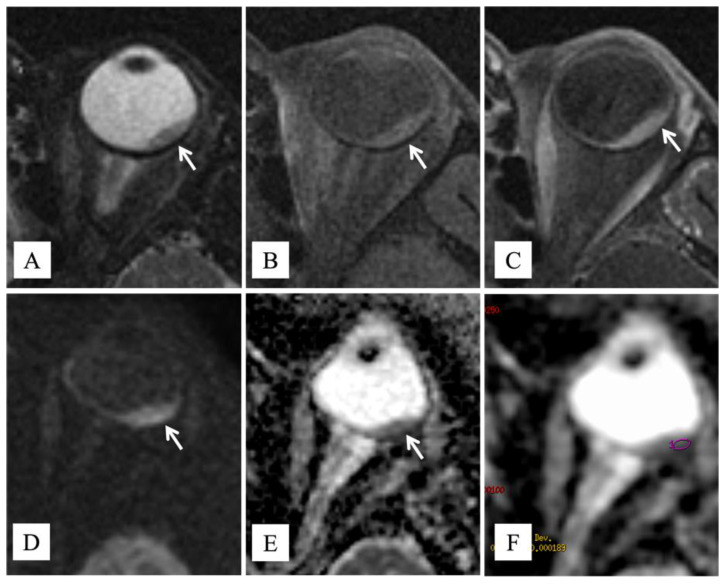
MR findings. Axial (**A**) T2-weighted turbo spin-echo STIR and (**B**) fat-suppressed T1-weighted images show a lentiform-shaped intraocular mass along the postero-lateral aspect of the left globe (white arrows), adjoining the optic disk. The lesion exhibits intermediate signal intensity on both T1- and T2-weighted images, a finding consistent with poorly pigmented melanoma. On (**C**) axial contrast-enhanced fat-suppressed T1-weighted image, the neoplasm demonstrates marked enhancement (white arrow). On (**D**) axial DW image (b = 1000 s/mm^2^) and (**E**) corresponding ADC map, the tumor displays restricted diffusion with high signal intensity on DWI image and low signal intensity on ADC map (white arrows), a finding consistent with hypercellularity. (**F**) The ADC value was 840 × 10^−6^ mm^2^/s.

A 69-year-old female patient was admitted to the Eye Clinic of our hospital for acute onset of visual disturbances. Ophthalmoscopic examination showed an amelanotic uveal melanoma involving the macula. A previous diagnosis of choroidal nevus was reported in 2019; at the examination, maximum diameter and thickness were 14.2 mm and 3 mm, respectively. The tumor presented lipofuscin and mild subretinal fluid. Fluorescein angiography showed progressive hyperfluorescence with late leakage. Magnetic resonance imaging (MRI) of the orbit showed a lentiform-shaped mass in the posterior chamber of the eye. The lesion demonstrated intermediate signal intensity on both T1- and T2-weighted images, restricted diffusion on diffusion-weighted imaging (DWI), and pronounced enhancement after intravenous administration of gadolinium-based contrast agent. The apparent diffusion coefficient (ADC) value of the lesion, measured on the ADC map derived from DW images, was 840 × 10^−6^ mm^2^/s. (Figure 1). These findings, in particular the intermediate signal intensity on T1- and T2-weighted sequences, were consistent with a poorly pigmented or amelanotic choroidal melanoma [12]. Neither metastases nor other primary tumors were found with total body Computed Tomography (CT) and hepatic echography.

**Figure 2:**
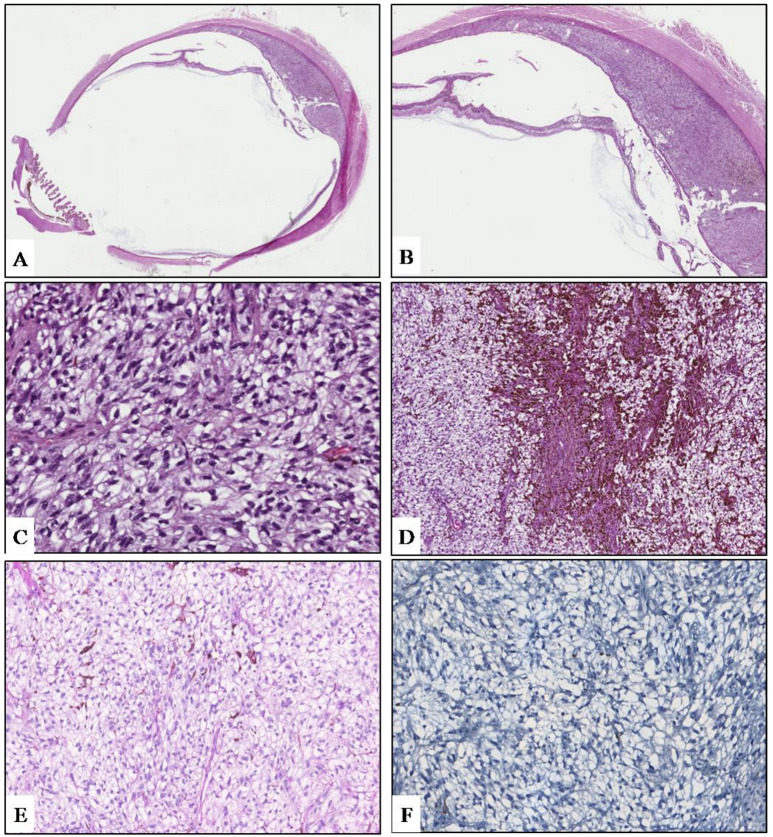
(**A**) Low magnification showing an ovoidal-shaped, poorly-pigmented choroidal tumor of the posterior ocular chamber (hematoxylin and eosin; original magnification 25×). (**B**) Due to the mass effect, the lesion induces a massive retinal detachment (hematoxylin and eosin; original magnification 50×). (**C**) Tumor exhibits a diffuse ballooning morphology, consisting of medium-sized, ovoidal cells with abundantly to weakly clear eosinophilic granular cytoplasm; few pigmented melanophages are also seen (hematoxylin and eosin; original magnification 400×). (**D**) A small portion of the neoplasm is composed of more pigmented spindle-shaped cells with elongated nuclei and conspicuous nucleoli (hematoxylin and eosin; original magnification 150×). Neoplastic cells are consistently negative for PAS (**E**) and BAP-1 (**F**) immunostaining. (**E**), periodic acid–Schiff staining; (**D**), immunoperoxidase; original magnifications 300×.

The diameter of the lesion, macular involvement that would not have allowed good post-treatment visual acuity, and the patient’s will led to the decision to perform eye enucleation. The surgical sample was fixed in neutral-buffered 10% formalin and submitted for histologic examination. On gross examination, a grey-whitish lesion measuring 1.6 × 0.3 cm was found on the cut surface. Histologically, low magnification showed a choroidal poorly pigmented tumor with sharply circumscribed margins (Figure 2A), protruding into the posterior segment of the eye, that induced a massive retinal detachment (Figure 2B). The tumor was mainly composed (90%) of small- to medium-sized, ovoidal cells with rounded to oval-shaped hyperchromatic nuclei with dispersed chromatin and inconspicuous nucleoli, and abundant clear or weakly eosinophilic cytoplasm, imparting cells a ballooning morphology (Figure 2C). The tumor also exhibited a rich vascular component, consisting of thin-walled small- to medium-sized vessels with a “branching” appearance. Few inflammatory cells, including lymphocytes and melanin-laden macrophages, were admixed to the neoplastic cells (Figure 2C). A small portion of the lesion (10%) showed a conventional spindle-cell morphology with elongated neoplastic cells with ovoidal nuclei and evident nucleoli (Figure 2D). Tumor cells were not stained with periodic acid−Schiff (PAS) reaction, revealing the lack of glycogen content (Figure 2E). Few mitoses (1/10 HPFs) were found. Atypical mitoses and necrosis were absent. No invasion of the sclera, ciliary bodies, and optic nerve was observed. Based on the above-mentioned morphological features, a diagnosis of “balloon cell melanoma of the choroid” was rendered. Based on tumor maximum diameter and thickness, the pathological T stage was pT2a. In order to investigate the molecular landscape of this tumor, molecular analyses were performed. No mutations were detected in *NRAS*, *BRAF*, and *GNAQ* genes, whereas we identified, according to the FATHMM score, the pathogenic *GNA11* p.Gln209Leu mutation and two neutral variants on *APC* and *TP53* genes (Table 1). In addition, tumor exhibited the immunohistochemical loss of BAP1, representing a surrogate of *BAP1* mutation (Figure 2F).

## 4. Discussion

Classically, UM is classified as spindle cell, epithelioid cell, or mixed cell type, with the epithelioid cell type being associated with a significantly worse prognosis. Among these conventional UM subtypes, some unusual histopathologic variants of UM have been reported, including oncocytic, neuroendocrine, signet ring cell, lipomatous, and balloon or clear cell. Although their prognostic significance is currently unclear, pathologists should be aware of these morphologic variants to avoid misdiagnosis with metastatic neoplasms [13]. Balloon cells can be observed in naevi and more rarely in malignant melanomas of the skin, conjunctiva, and uveal tract [14]. A study by Saornil et al. showed that balloon cell changes, along with necrosis and fibrosis, were more frequently found in choroidal melanomas that had been previously treated with proton beam irradiation and then enucleated, rather than in those whose primary treatment was the enucleation [9]. To classify a melanoma as a balloon cell variant, more than 50% of the neoplasm must be composed of balloon cells. There are no distinctive clinical features except that a polypoid gross appearance is more commonly seen in balloon cell variants. Prognosis is usually poor, and, as for the other UM variants, it is related to the pathological T stage rather than to this unusual morphology. Rodrigues et al. reported the clinicopathologic features of three patients affected by balloon cell melanoma of the choroid [15]. Ophthalmoscopically, small, slowly progressive tumors at the posterior pole, with an encircling yellow halo, were seen in all cases. On fluorescein angiography, the yellow halo showed a similar fluorescence to the remaining tumor, thus differentiating these morphologic changes from lipofuscin pigment, drusen, and exudates. Histologic examination demonstrated a prominent balloon cell component, which was more represented at the tumor margins. These cells exhibited variable melanin pigmentation and were negative for lipid, acid mucopolysaccharide, and glycogen. Special enzyme studies (lactic dehydrogenase, succinic dehydrogenase, beta-glucuronidase, acid phosphatase, and aminopeptidase) demonstrated some similarity to melanocytic cells [15]. Riley et al. reviewed 200 cases of malignant melanoma of the choroid and ciliary body, reporting that the incidence of balloon cell changes in these tumors was 10%, and that such a cell component was more commonly present in spindle cell-type tumors [10]. Khalil reported the clinicopathologic and ultrastructural findings in two cases of balloon cell UM. The primary biological alteration in the cell organelles leading to the formation of the balloon cells seemed to be the degradation of the melanosomes of the tumor cells, which determined the secondary increase in intracellular lipid secretion and accumulation. According to this author, although balloon cell changes were degenerative in nature and prevented necrosis of the tumor cells, distant metastases occurred in both cases, suggesting that the presence of balloon cells does not alter the prognosis of UM [11]. Jakobiec et al. described two cases of balloon cell melanomas of the ciliary body and, based on histochemical stains and electron microscopic observations, concluded that balloon cells in such cases represented spindled melanoma cells that had undergone extensive cytoplasmic lipid metamorphosis [16]. A comparative study by Heindl et al., which related the histological findings of 12 irradiated UMs to an equal number of control tumors, demonstrated that the irradiated melanomas showed significantly higher degrees of necrosis, balloon cell degeneration, and fibrosis than controls [17]. Another study by Messmer et al. concluded that the balloon cell changes, alongside the other degenerative changes showed by 56 irradiated Ums, were to be considered the result of radiation injury rather than spontaneous tumor regression when compared to 70 control eyes that had been enucleated without prior treatment for uveal melanoma [8]. In the present case, the tumor supposedly evolved from a choroidal nevus; no metastases were found, and the pathological T stage was pT2a; both ophthalmoscopic examination and conventional MRI T1- and T2-weighted sequences revealed morphological findings consistent with a poorly pigmented or amelanotic choroidal melanoma (lentiform shaped mass with intermediate signal intensity) [12]. We also performed DW sequences that have represented for years an integral part of our MR protocol. On DWI and the corresponding ADC map, the lesion was characterized by restricted diffusion; the ADC value, the quantitative parameter of diffusion, was 840 × 10^−6^ mm^2^/s. In previous reports, the mean ADC value of uveal melanomas ranges from 891 ± 172 × 10^−6^ mm^2^/s to 1180 ± 160 × 10^−6^ mm^2^/s [18]. DWI is an MR imaging technique, which provides information about the tissue cellularity and integrity of the cellular membranes and is able to quantify water diffusion in vivo through the measurement of ADC [19]. In our case, we found a lower ADC value than that previously reported and hypothesize that pathologic and ultrastructural findings of balloon cell melanoma, such as lack of necrosis, increase in intracellular lipid secretion and accumulation, and vacuolation in the cytoplasm, could be the reason for such a low ADC value [20,21]. Naturally, our observation deserves further investigations with larger series to verify the potential contribution of DWI in the characterization of uveal melanomas and their histologic subtypes.

## 5. Conclusions

Balloon cell melanoma of the choroid is a rare morphological variant of UM that prognostically might be no different from conventional UM. The literature provides few examples of this tumor, and in most of them, this morphology has been considered a degenerative change, secondary to proton beam irradiation.

In our opinion, the present case is particularly remarkable in that an extensive (90%) balloon cell morphology occurred in a UM primarily treated by enucleation of the eye; accordingly, we believe that, in our case, balloon cell morphology was an intrinsic feature of the tumor and not a degenerative cell reaction. Histopathologic differential diagnosis of balloon cell UM mainly includes ocular metastasis from clear cell neoplasms (i.e., renal clear cell carcinoma) and balloon cell choroidal nevus. In the present case, the former has been excluded based on the absence of other primary tumors on total body CT, but it could also be excluded by the immunohistochemical positivity for melanocytic markers, such as SOX10, Melan-A, S-100, and HBM-45, whereas the latter has not been taken into account due to the presence of overtly malignant histopathologic features.

Functional MRI techniques, such as DWI with quantitative measurement of ADC, could be a useful diagnostic tool [19], whereas ophthalmoscopic examination is often non-specific, showing slowly enlarging, poorly pigmented choroidal lesions with encircling yellow halo [15].

## Figures and Tables

**Table 1:** Variants detected by next generation sequencing.

Locus	Genes	Ref	Alt	AF %	Coding	AA Change	FATHMM
chr5:112175769	APC	G	A	45.28	c.4479G > A	p.Thr1493=	Neutral (score 0.46)
chr17:7579472	TP53	G	C	94.79	c.215C > G	p.Pro72Arg	Neutral (score 0.36)
chr19:3118942	GNA11	A	T	34.92	c.626A > T	p.Gln209Leu	Pathogenic (score 0.98)

Abbreviations: Ref: reference sequence; Alt: alternative sequence; AF: allele frequency; AA: amino acid.

## Data Availability

No new data were generated in this study.
